# Novel subcellular localization for *α*-synuclein: possible functional consequences

**DOI:** 10.3389/fnana.2015.00017

**Published:** 2015-02-23

**Authors:** Cristina Guardia-Laguarta, Estela Area-Gomez, Eric A. Schon, Serge Przedborski

**Affiliations:** ^1^Departments of Pathology, Columbia University Medical CenterNew York, NY, USA; ^2^Departments of Neurology, Columbia University Medical CenterNew York, NY, USA; ^3^Departments of Genetics and Development, Columbia University Medical CenterNew York, NY, USA

**Keywords:** alpha-synuclein, Parkinson’s disease, mitochondria-associated membranes, endoplasmic reticulum, phospholipid

## Abstract

α-synuclein (α-syn) is one of the genes that when mutated or overexpressed causes Parkinson’s Disease (PD). Initially, it was described as a synaptic terminal protein and later was found to be localized at mitochondria. Mitochondria-associated membranes (MAM) have emerged as a central endoplasmic reticulum (ER) subcellular compartments where key functions of the cell occur. These domains, enriched in cholesterol and anionic phospholipids, are where calcium homeostasis, lipid transfer, and cholesterol metabolism are regulated. Some proteins, related to mitochondrial dynamics and function, are also localized to this area. Several neurodegenerative diseases have shown alterations in MAM functions and resident proteins, including Charcot Marie-Tooth and Alzheimer’s disease (AD). We have recently reported that MAM function is downregulated in cell and mouse models of PD expressing pathogenic mutations of α-syn. This review focuses on the possible role of α-syn in these cellular domains and the early pathogenic features of PD that could be explained by α-syn-MAM disturbances.

## Parkinson disease background

Parkinson disease (PD) is the second most prevalent neurodegenerative disease after Alzheimer disease (AD). Its main symptoms are resting tremors, rigidity, slowness of voluntary movements, freezing, and postural instability. Histopathologically, this disease is characterized by (a) a significant loss of dopaminergic neurons in the substantia nigra pars compacta (SNpc; Braak et al., 2003) and (b) the accumulation of intracytoplasmic aggregates called Lewy bodies, composed mainly of alpha-synuclein protein (α-syn; Spillantini et al., 1997, 1998). This aggregation occurs at the SNpc and other cerebral areas such as locus ceruleus, nucleus basalis, hypothalamus, cerebral cortex, and autonomic nervous system (Maroteaux et al., 1988; Parkinson, 2002).

The majority of the PD cases are sporadic with only less than 10% of the cases related to mutations in genes such as *PARK2*, *PARK7*, *PINK1*, *LRRK2* or *SNCA* (Polymeropoulos et al., 1997; Krüger et al., 1998; Zarranz et al., 2004). Among these, mutations or duplication in *SNCA*, which codifies for α-syn, have been shown to cause autosomal dominant forms of familial PD (Krüger et al., 1998; Singleton et al., 2003; Zarranz et al., 2004; Schon and Przedborski, 2011).

## Subcellular localization α-synuclein

α-syn is a 140 aa protein, highly expressed in nervous tissues, that was identified as the precursor protein for the non–beta amyloid component of AD plaques (Uéda et al., 1993). Despite numerous research efforts, its main function remains unknown.

The majority of α-syn is soluble and resides in the cytoplasm. However, many researchers have demonstrated that α-syn, upon a yet unknown stimulus, is capable of binding to membranes and changes its N-terminal domain conformation upon this interaction (Eliezer et al., 2001; Jao et al., 2004, 2008). *In vitro*, α-syn binds preferentially to anionic phospholipids and liposomes of high curvature (Davidson et al., 1998; Fortin et al., 2004; Auluck et al., 2010). In the cell, these membrane regions are called lipid raft domains, which are detergent resistant membranes (DRM) with unique molecular characteristics (Simons and Toomre, 2000). Initially, lipid rafts were believed to form only at the plasma membrane; however, many authors have shown that these domains can also be localized intracellularly (Hayashi and Fujimoto, 2010).

In an effort to understand the function of this protein, many groups have reported several subcellular localizations for α-syn. In the last decades, multiple research data have shown α-syn located at pre-synaptic terminals (Kahle et al., 2000), participating in the regulation of the synaptic pool size and neurotransmitter release (Iwai et al., 1995; Masliah et al., 1996; Abeliovich et al., 2000; Murphy et al., 2000; Cabin et al., 2002; Gitler et al., 2008).

More recently, α-syn has been reported to bind to mitochondria (Li et al., 2007; Cole et al., 2008; Devi et al., 2008; Parihar et al., 2008; Zhang et al., 2008). This binding is especially significant in the striatum, substantia nigra (SNpc), and cortex of PD brains (Devi et al., 2008). Supporting these results, a recent study describes the existence of an N-terminal sequence in α-syn that could work as a mitochondrial targeting sequence (Devi et al., 2008). Moreover, α-syn binding to membranes is favored by the presence of cardiolipin (Zigoneanu et al., 2012), a lipid specific of the mitochondria membrane.

## Mitochondria and α-synuclein in the pathogenesis of PD

Supporting α-syn localization to the mitochondria, PD patients, and cellular models containing pathogenic mutations of this protein show a deficit in mitochondrial functionality (Hsu et al., 2000; Schon and Przedborski, 2011), and, in particular, a significant decrease in complex I activity (Devi et al., 2008). In fact, the decrease in complex I activity is also present in PD brains and cellular models containing mutations in other genes related to the disease.

Moreover, exposure to a contaminant called 1-methyl-4-phenyl-1,2,3,4-tetrahydropyridine (MPTP), which is an inhibitor of the mitochondrial complex I, provokes parkinsonism symptoms and loss of dopaminergic neurons (Langston et al., 1983; Dauer and Przedborski, 2003). Additionally, injection of another complex I inhibitor, rotenone, caused a similar phenotype (Betarbet et al., 2000). All these data supports a role for mitochondrial dysfunction in the pathogenesis of PD.

In addition to complex I dysfunction, pathogenic mutations of α-syn have also been shown to interact with and reduce the activity of complex IV (Elkon et al., 2002). Also, there is data that relates the age-related accumulation of non-aggregated α-syn to mitochondria with a reduced dopamine phenotype in the SNpc (Chu and Kordower, 2007). Studies in one of the transgenic models of PD bearing the α-syn A53T mutation, show not only complex I inhibition, but also damaged mitochondrial DNA and aberrant mitochondrial dynamics (Martin et al., 2006; Chinta et al., 2010; Choubey et al., 2011).

It is widely known that mitochondria are dynamic organelles that undergo fusion and fission continuously (Chan, 2006). Mitochondrial movement is especially dramatic in neurons, where mitochondria travel along the axons to provide the terminals with ATP and other metabolites. Perturbations of this flux of mitochondria throughout the cell body cause defects in cell viability. Curiously, alterations in mitochondrial dynamics have been extensively reported in numerous neurodegenerative diseases, i.e., AD (Wang et al., 2008, 2009), PD (Yu et al., 2011; Cooper et al., 2012) and Charcot Marie-Tooth (Baloh et al., 2007; Chen and Chan, 2009). For instance, in the case of PD, the mitochondrial protein PINK1, whose mutation causes PD, is known to interact with the proteins Miro and Milton, both microtubule-associated proteins (Weihofen et al., 2009). Moreover, research data showed that aggregates of wild-type α-syn disrupt the mitochondrial trafficking of cargoes (Galvin et al., 1999; Lee et al., 2006).

An alternative consequence of the deregulation of mitochondrial trafficking is the alteration of the mitochondrial quality control. Spare or damaged mitochondria are degraded by mitophagy, a process in which the cell has to be able to differentiate healthy from damaged mitochondria. An indication of healthy mitochondria is a high membrane potential and low reactive oxygen species (Twig and Shirihai, 2011). The primary mechanism for degrading or minimizing damaged mitochondria is fusion and fission (Schon and Przedborski, 2011). During these processes, the cell “neutralize” unhealthy mitochondrial content, mixing it with other healthy organelles. In the case of neurodegenerative diseases, such as PD, where the fusion/fission machinery is altered, the accumulation of “bad” mitochondria can lead to the disease. More specifically, some authors link those dysfunctional mitochondria that cannot reach axonal extremes in PD with an increased expression of α-syn and aggregation (Lee et al., 2002). This could be the cause of the accumulation of mitochondrial mutations observed in the SNpc of PD patients that leads to a loss of dopaminergic neurons (Bender et al., 2006; Kraytsberg et al., 2006).

## α-synuclein is localized at mitochondria-associated membranes

Trying to answer this question, we revisited the exact cellular localization of α-syn. We have recently described a more accurate localization of α-syn (Guardia-Laguarta et al., 2014). Our data shows the existence of a subpopulation of α-syn that resides at the mitochondria-associated membranes or MAM. This region interconnects the endoplasmic reticulum (ER) and the mitochondria and is responsible for specific cellular functions. These membranes are composed of intracellular lipid rafts. This result is compelling, as previous works show that α-syn has an affinity for lipid rafts (Fortin et al., 2004) and negatively-charged membranes (Davidson et al., 1998) and could possibly explain the studies describing α-syn as a mitochondrial protein (Li et al., 2007; Cole et al., 2008; Devi et al., 2008; Parihar et al., 2008; Shavali et al., 2008). The lack of appropriate markers for MAM and the technical difficulty in fractionating this kind of membrane because of its association with the ER could explain previous results (Area-Gomez et al., 2012).

**Figure 1:**
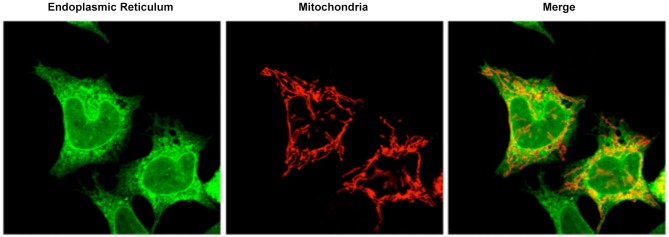
**Confocal microscopy image representative of the ER mitochondrial connections in M17 dopaminergic cell line.** ER is labeled with GFP-Sec61-β (green) and mitochondria labeled with pDsRed2-mito (red). Merge image indicates the colocalization of both organelles.

MAM is a subcompartment of the ER that is connected to the mitochondria (Figure 1; Rusiñol et al., 1994; Csordás et al., 2006; Hayashi et al., 2009). It is involved in a number of core cellular functions; i.e., calcium homeostasis (Csordás et al., 2010), cholesterol metabolism (Rusiñol et al., 1994), and phospholipid transfer from the ER to the mitochondria (Vance, 1990). Specifically, MAM has been described as the residence of several proteins related to phospholipid regulation (phosphatidylserine synthase 2: PTDSS2), cholesterol metabolism (acyl-CoA:cholesterol acyltransferase) (Rusiñol et al., 1994), and calcium transport from the ER to the mitochondria (the type 3 inositol 1,4,5-triphosphate receptor, IP3R3) (Hayashi and Fujimoto, 2010). Notably, mitochondrial distribution and dynamics are influenced by the physical connections formed by MAM (Rizzuto et al., 1998; Levine and Rabouille, 2005; Csordás et al., 2006; Hayashi et al., 2009; Friedman et al., 2011; Rowland and Voeltz, 2012). During mitochondrial fission, ER tubules appear to “embrace” mitochondria and mark sites of mitochondrial division (Friedman et al., 2011). In addition, isolated MAM from different tissues have been shown to be enriched in proteins related to the control of mitochondrial dynamics (e.g., FIS1, MFN2, and DRP1). Finally, MAM also contain some proteins involved in apoptosis (e.g., VDAC1 [voltage-dependent anion channel 1], BAX and BID (Garofalo et al., 2005; Ciarlo et al., 2010)). Indeed, calcium release at ER-mitochondrial contacts, which is important for ATP production, could be responsible for sensitizing mitochondria to apoptosis (Iwasawa et al., 2011; Tabas and Ron, 2011). The alteration of mitochondrial-ER contacts can cause deregulation of the calcium signal which results in inappropriate protein folding, metabolic alterations, and apoptosis (Csordás and Hajnóczky, 2009; Bui et al., 2010).

## Could the pathogenesis of PD be explained by early alteration in MAM function?

Scorrano’s group made the first correlation between MAM disturbance and disease when they described Mfn2 as a MAM resident protein that participates as a scaffold between ER and mitochondria. Mutations of Mfn2 cause Charcot Marie Tooth type 2a (de Brito and Scorrano, 2008). More recently it has been shown that presenilin-1, presenilin-2, and γ-secretase activity—all key factors associated with the pathogenicity of AD—are highly enriched in the MAM (Area-Gomez et al., 2009). Moreover, mutation or ablation of these γ-secretase components provokes a significant upregulation of several activities located at the MAM (Area-Gomez et al., 2012). Similarly, we have also shown that mutations in α-syn cause an alteration in the regulation of MAM function (Guardia-Laguarta et al., 2014). Supporting this observation, several groups have reported alterations in the lipidic composition of membranes from PD brains (Fabelo et al., 2011). These data suggest that these molecular alterations would change thermodynamic properties, organization, and signal transduction in the PD brain.

MAM regulates the homeostasis of cholesterol through the acyl-coA cholesterol acyltransferase (ACAT) activity. ACAT is the enzyme responsible of the conversion of free cholesterol to cholesteryl esters that eventually will be stored as lipid droplets. Therefore, ACAT activity regulates the amount of free cholesterol in cellular membranes. Cholesterol regulation alterations in PD have been extensively reported in the literature (de Lau et al., 2006; Huang et al., 2007; Hu et al., 2008). Interestingly, α-syn contains two cholesterol binding domains that play a role in the regulation of its binding to membranes and perhaps aggregation (Fantini and Yahi, 2013). In fact, α-syn transgenic mice treated with statins (cholesterol-lowering drug) showed a significant reduction in α-syn aggregation (Bar-On et al., 2008). Finally, numerous reports have described the interaction of α-syn and lipid droplets (Cole et al., 2002; De Franceschi et al., 2009). Taking all of this into account, it may well be that the cholesterol alterations in PD are a consequence of a MAM dysfunction caused by mutations in α-syn, a MAM protein.

The transfer of calcium between ER and mitochondria via MAM is a highly regulated process that controls the whole calcium homeostasis in the cell (Rizzuto et al., 2009; Csordás et al., 2010). As in many other neurodegenerative diseases, calcium homeostasis is altered in PD patients and animal models. In neurons, these alterations result in excitotoxic events that may eventually cause cell death (Rizzuto et al., 2009). Brini’s group was the first to show that α-syn is involved in the regulation of calcium homeostasis by altering the ER-mitochondria communication (Calì et al., 2012). Hodge and Colombini (1997) show that VDAC, a voltage-dependent calcium channel that controls mitochondrial calcium levels and mitochondrial function (and is localized in MAM), is decreased in nigral neurons positive for α-syn. Finally, recent evidence shows that increased Parkin expression improves calcium transfer through MAM; implying that Parkin mutations that cause PD could be detrimental for maintaining healthy levels of calcium (Calì et al., 2013).

Oxidative stress has been considered one of the main factors in the pathogenesis of PD (Kidd, 2000; Jenner, 2003). Increased levels of lipid hydroperoxydes have been found in SNpc and midbrain from PD patients (Yoritaka et al., 1996). Indeed, oxidative damage, lipoxidation of proteins like α-syn and oxidative DNA damage have been found in early-stages of PD (Dalfó et al., 2005), indicating a role for oxidative stress in the disease. As a response to this insult, it has been suggested that the SNpc suffers an increase in the turnover of membrane phospholipid synthesis that may be behind the specificity of neuronal death in the SNpc in PD (Ross et al., 2001). Again, the results point to an early imbalance in a very basic function of the cell, as it is the phospholipid transfer, controlled by MAM membranes that, over time, cause a disabling neurodegenerative process.

It is also well known that ER-mitochondria connections regulate mitochondrial dynamic processes (Csordás et al., 2006; Hayashi et al., 2009; Friedman et al., 2011; Rowland and Voeltz, 2012). It has been previously described that mutations in α-syn increase mitochondrial fragmentation (Kamp et al., 2010; Nakamura et al., 2011). Correlating this to MAM dysfunction, we have confirmed this fragmented phenotype in our mutant cells (Guardia-Laguarta et al., 2014). Nevertheless, it is possible that the fragmentation observed when α-syn is mutated is not due to defects on the fusion/fission mitochondrial machinery but rather to MAM alteration (Guardia-Laguarta et al., 2014).

Next, autophagy, a strictly regulated mechanism, is altered in PD (Chinta et al., 2010). Actually, PINK1 and Parkin, are known to be part of the mitochondrial autophagy cascade, or mitophagy. There is also evidence of the relation of α-syn with mitophagy: first it was described that transgenic animals expressing A53T α-syn present alterations in mitophagy (Chinta et al., 2010). The same result was found in yeast expressing wild-type α-syn (Sampaio-Marques et al., 2012). Interestingly, it has been reported that the autophagosomes, a key step during autophagy pathway, are formed at the MAM boundaries (Hamasaki et al., 2013).

Finally, consistent with other authors (Calì et al., 2012), our results suggest that pathogenic mutations result in a lower binding of α-syn to MAM. Therefore, it is possible that a certain amount of wild-type α-syn is necessary to maintain normal function, and so, mutation in α-syn does not cause a toxic gain-of-function, but rather a loss of “relevant” function in mitochondrial morphology maintenance and in some of the main MAM functions.

## Future questions

While we believe that our data help create a new way of thinking about PD pathogenesis, many questions need further research to properly address this new “MAM hypothesis”.

First, α-syn was initially described as a protein with a perinuclear and pre-synaptic dual localization, hence the name. The perinuclear localization is in agreement with our data because MAM, as part of the ER, is known to be enriched around the nucleus (de Brito and Scorrano, 2010). However, in order to satisfy both the pre-synaptic and this new MAM localization, the ER-mitochondria domains should also be present at these synaptic terminals. Notably, some authors have shown that ER-mitochondrial connections (McNulty, 1980) and known MAM markers (Mavlyutov et al., 2012) can be found closely juxtaposed to synaptic membranes. Moreover, similar connections between ER and mitochondria were also observed in ganglion cell membranes close to nerve endings (Watanabe and Burnstock, 1976; Taxi and Eugène, 1995).

Second, our data does not address the question of whether the toxic effect of α-syn is due to its aggregation tendency or to its soluble state, or caused by the overexpression of the protein (Narhi et al., 1999; Goldberg and Lansbury, 2000; Ostrerova-Golts et al., 2000). Furthermore, none of the experiments carried out reveal any aggregation of α-syn in dopaminergic cells lines or tissues. It may well be that only monomeric α-syn in MAM initiate the cascade of events that ultimately leads to mitochondrial dysfunction and dopaminergic cell loss. Equally possible is that α-syn binding to MAM triggers the aggregation of this protein into oligomers.

Finally, our work focuses only on the relationship between α-syn and MAM. Whether MAM dysfunction is an event also related to mutations in other PD genes requires additional investigation. Nonetheless, cellular symptoms caused by pathogenic mutations in these other genes are practically identical to those provoked by mutations in α-syn.

## Conclusions

Our data suggest that MAM alteration may play an important role during the progression of PD pathology as an early event that may cause an imbalance in basic functions of the cell. Our “MAM hypothesis” helps reconcile many of the cellular symptoms seen in PD over time, such the accumulation of unhealthy mitochondria, altered autophagy, dysfunctional calcium levels, increased lipid droplets, and altered phospholipid species that lead to neurodegeneration. In addition, the localization of α-syn in MAM may help reconcile questions regarding the role of both the ER and mitochondria in the pathogenesis of PD, and may explain some of the features of DA neuron degeneration, i.e.: the deregulation of calcium homeostasis and mitochondrial dysfunction.

We hypothesize that a more detailed study of other functions that are located in MAM will reveal other alterations related to PD progression and that MAM alteration could be a good pre-symptomatic predictor of future PD pathology.

## Conflict of interest statement

The authors declare that the research was conducted in the absence of any commercial or financial relationships that could be construed as a potential conflict of interest.
